# Paxlovid for the treatment of severe or critical COVID-19 in children

**DOI:** 10.1186/s12887-025-05807-1

**Published:** 2025-07-02

**Authors:** Linjuan Xiang, Qun Wang, Yanwen Xu, Yu Tong, Yuhang Wu, Xiaoshan Zhang, Xinxin Zeng, Sheng Ye, Chenmei Zhang, Linhua Tan, Lvchang Zhu, Jing Miao, Sun Chen, Xi Zhang, Xuben Yu, Lisu Huang

**Affiliations:** 1 Department of Infectious Disease, Children’s Hospital, School of Medicine, Zhejiang University, National Clinical Research Center for Child Health, Hangzhou, 310052 China; 2 Department of Infectious Disease, Xinhua Hospital, Shanghai Jiao Tong University School of Medicine, Shanghai, 200092 China; 3 Department of Pharmacy, The First Affiliated Hospital of Wenzhou Medical University, Wenzhou, 325000 China; 4 Department of Pediatric Intensive Care Unit, Children’s Hospital, Zhejiang University School of Medicine, National Clinical Research Center for Child Health, Hangzhou, 310052 China; 5 Department of Surgical Intensive Care Unit, Children’s Hospital, Zhejiang University School of Medicine, National Clinical Research Center for Child Health, Hangzhou, 310052 China; 6 Department of Pharmacy, Children’s Hospital, Zhejiang University School of Medicine, National Clinical Research Center for Child Health, Hangzhou, 310052 China; 7 Department of Pediatric Cardiology, Xinhua Hospital, Shanghai Jiao Tong University School of Medicine, Shanghai, 200092 China; 8 Clinical Research Unit, Xinhua Hospital, Shanghai Jiao Tong University School of Medicine, Shanghai, 200092 China

**Keywords:** COVID-19, Children, Critical, Paxlovid, Pharmacokinetics

## Abstract

**Background:**

Paxlovid, known for its efficacy against SARS-CoV-2, is currently limited in its use for treating pediatric COVID-19, particularly in severe or critical cases.

**Methods:**

We conducted a study within a single-center, prospective cohort of 450 children diagnosed with COVID-19 between December 2022 and May 2023. This study included 30 pediatric patients who received Paxlovid and 60 matched controls who did not, based on factors such as age, disease severity, and underlying health conditions. Safety was assessed through the incidence of adverse events, and laboratory parameters. The time to clinical symptom improvement was the main efficacy outcome. Moreover, we calculated the AUC_0 − 12 h_ of Nirmatrelvir of the Paxlovid patients.

**Results:**

Adverse events occurred in 16.7% of both groups, with no serious events reported. The Paxlovid group showed a significantly shorter time to viral clearance, fever resolution, and symptom recovery compared to controls (4.9 vs. 11.0 days, *P* = 0.01; 11.2 vs. 16.4 days, *P* = 0.01; 4.6 vs. 17.6 days, *P* < 0.01). This effect was most noticeable in children with underlying conditions or those treated early. No significant differences were observed in ICU transfers or mortality (*P* > 0.05). The AUC₀-₁₂_h_ of Nirmatrelvir did not significantly alter treatment outcomes.

**Conclusion:**

Our findings suggest that Paxlovid may be a safe and effective option for treating severe or critical COVID-19 in children.

**Supplementary Information:**

The online version contains supplementary material available at 10.1186/s12887-025-05807-1.

## Introduction

The COVID-19 pandemic brought over 774 million infections and 7 million fatalities worldwide [1]. Up to now, there are still 200–300 thousand additional cases and several hundred deaths per week across the globe. Nirmatrelvir/Ritonavir (Paxlovid) has been proven effective for the treatment of COVID-19 in adults [2]. However, clinical data on its use in children remain limited. While Phase II/III trials for pediatric patients (NCT05261139) are ongoing, no published results are yet available. Only a few case reports and small case series have assessed Paxlovid in children [3–6], with one retrospective study evaluating its safety and efficacy in 31 pediatric ICU patients, including 9 who received Paxlovid [4, 7–9]. Due to the lack of robust evidence [10, 11], current guidelines recommend the use of Paxlovid in children on a case-by-case basis. This uncertainty can present challenges for clinicians in making treatment decisions [12, 13].

Although Paxlovid has been recommended for the treatment of mild to moderate COVID-19 in adults, specifically when administered within 5 days of symptom onset [14], its safety and efficacy have not been evaluated in severe or critical cases [2, 15–19]. While COVID-19 in children is generally less severe than in adults [20–22], children with underlying health conditions are at a higher risk for complications such as hospitalization, intensive care, mechanical ventilation, and even death [23–27]. A global study involving 1,500 children with cancer found that 20% experienced severe or critical COVID-19, with a mortality rate of 3% [28]. Given that an estimated 400,000 children and adolescents aged 0–19 are diagnosed with cancer annually [29, 30], this suggests that approximately 12,000 children could potentially die from COVID-19. Therefore, providing Paxlovid to children, especially those with compromised immune systems due to underlying conditions, may help prevent the progression of COVID-19 to severe stages and reduce mortality rates [11].

To further assess the safety and effectiveness of Paxlovid in pediatric populations, we conducted a prospective observational study. This study included children and adolescents treated with Paxlovid, with cases stratified by severity, and followed up over a 6-month period.

## Methods

### Patients and setting

**Fig. 1:**
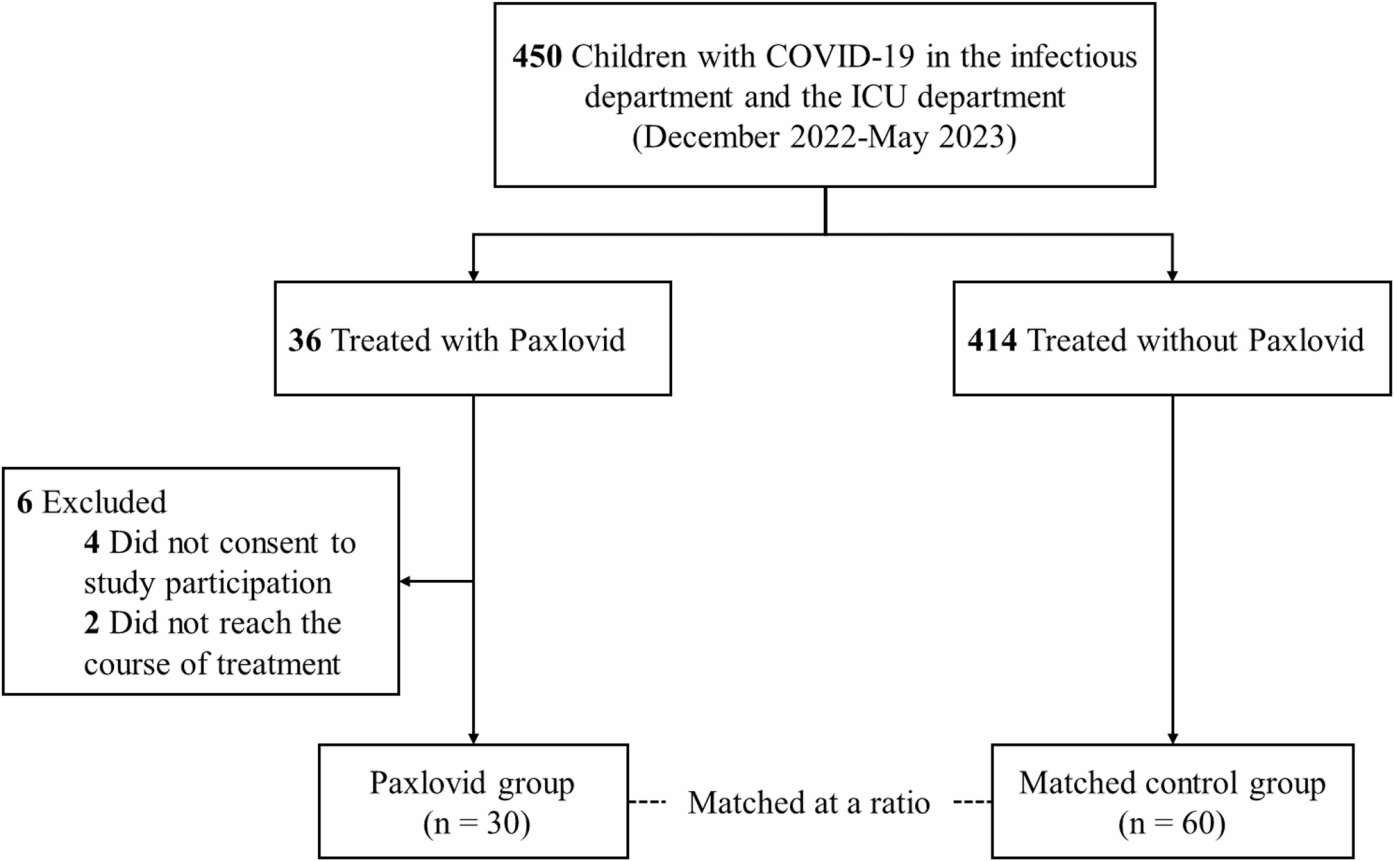
Flow chart

This prospective observational study was conducted within a single-center cohort of pediatric patients with infections (IRB No. 2023-IRB-0176-P-01). The study took place at the infectious department and the ICU department of the Children’s Hospital of Zhejiang University School of Medicine, the largest children’s hospital in East China, with over 3,500 beds and an annual outpatient volume exceeding 4 million visits. We reviewed the medical records of hospitalized patients aged 0 to 18 years who were diagnosed with COVID-19, confirmed by either nucleic acid or antigen test, between December 2022 and May 2023. Among the 450 children in the cohort, 36 received Paxlovid treatment. Patients who did not provide informed consent were excluded, resulting in 30 children in the Paxlovid treatment group (Fig. 1). A control group was formed by selecting children who did not receive Paxlovid, matched by age and underlying conditions. Each patient in the Paxlovid group was matched to two controls from the same period, based on similar age (within ± 2 years), disease severity (severe or critical COVID-19), and underlying medical conditions. The matching process followed the chronological order of patient admission, with only the first two patients admitted to the hospital being considered for matching in the control group. We also evaluated the exposure to Nirmatrelvir in these patients and examined the relationship between drug exposure, treatment efficacy, and adverse events.

### Treatment regimens

The indications, dosage, and administration frequency for Paxlovid were determined by a team of pediatricians and pharmacologists on a case-by-case basis, considering pediatric pharmacokinetic characteristics [31, 32]. It is important to note that our study focused on observation and data collection rather than active intervention. The recommended treatment regimen is as follows for children under 12 years old who weigh less 40 kg, the prescribed dosage is 7.5 mg/kg of Nirmatrelvir and 2.5 mg/kg of Ritonavir, administered twice daily for five days. For children aged 12 to 18 who weigh more than 40 kg, the dosage is 300/100 mg of Nirmatrelvir/Ritonavir orally twice daily. In the control group, 60 patients received routine treatment. Further details regarding the Paxlovid dosing protocol can be found in the supplementary materials (Appendix S5). To distinguish between drug side effects and symptoms of the disease itself, adverse events were defined specifically as new clinical manifestations that arose after the administration of Paxlovid, as outlined in the Paxlovid package insert. Additionally, a qualified pharmacologist ensured that any potential adverse reactions from concurrent medications were excluded from the analysis.

### Data collection and definitions

Data were collected from patient medical records, including information on age, sex, weight, height, underlying conditions and laboratory parameters. Additional data included clinical symptoms at admission, recovery times for symptoms (excluding fever), adverse events during treatment and other relevant clinical details. Follow-up data were also gathered six months post-treatment, covering a range of symptoms and restoring academic activities. Serum concentrations were measured using liquid chromatography-tandem mass spectrometry (LC-MS/MS) (Supplementary Appendix S2). Blood samples were collected during treatment, whenever feasible.

### Time to negative conversion and primary efficacy endpoint

The time to negative conversion was defined as the period from the first dose of medication to the date of the first negative nucleic acid test (Supplementary Appendix S1). The primary efficacy endpoint was the time to recover symptoms (except fever), defined as the time from the first dose until all 13 COVID-19-related symptoms (listed in Table 1) were resolved, as assessed by the treating physicians. Disease severity was classified according to the Guidelines on the Diagnosis and Treatment of Corona Virus Disease 2019 (Tenth Trial Edition), issued by the National Health Commission and State Administration of Traditional Chinese Medicine (Supplementary Appendix S3). Underlying conditions include malignancies, cerebral palsy, leukemia, lymphoma, congenital heart defects, post-bone marrow transplantation status and renal failure.

**Table 1:** Baseline characteristics in the paxlovid group and matched control group in children with severe or critical COVID-19

Characteristics	Paxlovid group (*n* = 30)	Matched control group (*n* = 60)	*P*-value
**Age**,** year**,** mean ± SD**	7.5 ± 4.6	7.1 ± 4.9	0.72
**Male Gender**,** n (%)**	19 (63.3)	36 (60.0)	0.76
**BMI**,** kg/m**^**2**^, **mean ± SD**	16.9 ± 5.1	17.1 ± 5.0	0.66
**Underlying conditions**,** n (%)**	17 (56.7)	34 (56.7)	1.00
**Clinical symptoms at admission**,** n (%)**			
Fever	29 (96.7)	57 (95.0)	1.00
Coughing	17 (56.7)	44 (73.3)	0.11
Sputum	13 (43.3)	30 (50.0)	0.55
Wheezing	1 (3.3)	3 (5.0)	1.00
Tachypnea	9 (30.0)	12 (20.0)	0.29
Rash	4 (13.3)	3 (5.0)	0.22
Convulsions	5 (16.7)	11 (18.3)	0.85
Headaches	3 (10.0)	3 (5.0)	0.40
Drowsiness	3 (10.0)	2 (3.3)	0.33
Myalgia	2 (6.7)	3 (5.0)	1.00
Abdominal pain	4 (13.3)	5 (8.3)	0.47
Diarrhea	3 (10.0)	2 (3.3)	0.33
Vomiting	6 (20.0)	9 (15.0)	0.55
Dehydration	1 (3.3)	0 (0.0)	0.33
**Fever peak**,** mean ± SD**	39.1 ± 1.1	39.2 ± 0.9	0.67
**COVID-19 clinical types**,** n (%)**			0.66
Severe	14 (46.7)	31 (51.7)	
Critical	16 (53.3)	29 (48.3)	
**pSOFA score ≥ 2**,** n (%)**	16 (55.2)	18 (30.5)	0.03
**SIRS score ≥ 2**,** n (%)**	27 (93.1)	55 (91.7)	1.00

BMI, body mass index; Psofa, pediatric sequential organ failure assessment; SIRS, systemic inflammatory response syndrome; COVID-19, Corona Virus Disease 2019

### Assessment of clinical and laboratory parameters

Physicians evaluated and documented pre-existing conditions, symptoms, symptom resolution, as well as sores on the pediatric Sequential Organ Failure Assessment (pSOFA) [33] and Systemic Inflammatory Response Syndrome (SIRS) [34] scales. These clinical parameters were used to monitor disease progression and treatment response.

### Statistics analysis

Continuous variables were presented as means and standard deviation (SD) or medians and interquartile range (IQR), as appropriate. The Wilcoxon signed-rank test was used to evaluate the differences between the Paxlovid group and matched control group. Univariate analyses were performed using Cox proportional hazards regression to calculate hazard ratios. All statistical tests were two-tailed, and a *P* value of < 0.05 was considered statistically significant. Statistical analyses were conducted using R software version 4.3.1 (http://www.R-project.org) and Empower R(www.empowerstats.com, X&Y Solutions, Inc., Boston MA, USA).

Pharmacokinetic data for Nirmatrelvir and Ritonavir are presented in Supplementary Appendix S4. Time to negative conversion, fever duration and symptom recovery times were analyzed using Kaplan–Meier methods. Additionally, the clinical outcomes of fever and symptom duration (≤ 3 days) were compared with data from pediatric patients in the same region [35].

## Results

### Subject characteristics

A total of 90 patients were included in the study, and their baseline characteristics were generally similar across both groups, except higher pSOFA scores in the Paxlovid group compared to the control group (Table 1). During the six-month follow-up, one patient in the Paxlovid group developed seizures after encephalitis, and one patient in each group experienced SARS-CoV2 RT-PCR rebound. Additionally, eight patients had recurrent respiratory symptoms (3 [10.3%] in the Paxlovid group vs. 5 [8.5%] in the control group).

Regarding ICU admissions during hospitalization, one patient in the Paxlovid group and five patients in the control group were transferred (3.3% vs. 8.3%, *P* = 0.66). Mortality rates were similar between the groups during hospitalization, with 2 deaths (6.7%) in the Paxlovid group and 4 deaths (6.7%) in the control group. One month after discharge, the mortality rate increased to 13.3% (4 deaths) in the Paxlovid group and 8.3% (5 deaths) in the control group (*P* = 0.47). Over the course of the six-month follow-up, one patient from each group was lost to follow-up, resulting in a final mortality rate of 13.8% in the Paxlovid group and 11.9% in the control group (*P* = 0.82). No significant difference in mortality was observed between the groups (*P* > 0.05).

### Safety assessment

Both the Paxlovid and the control group had a similar overall incidence of adverse events during treatment (16.7% in both groups) (Table 2). There were no serious adverse events, and treatment with Paxlovid was generally well tolerated, with no treatment discontinuations. Among pediatric patients aged 6–11 years, the frequency of adverse events was similar between the Paxlovid and control groups (23.5% vs. 24.0%, respectively) (Supplementary Tables 1, 2). For patients under 6 years old, the control group had a slightly higher rate of adverse events compared to the Paxlovid group, although this difference was not statistically significant (0.0% vs. 17.4%, *P* = 0.29).

**Table 2:** Drug adverse events in the paxlovid group and matched control group

Variables, *n* (%)	Paxlovid group (*n* = 30)	Matched control group (*n* = 60)	*P*-value
**Adverse events**			
Diarrhea	1 (3.3)	3(5.0)	1.00
Vomiting	1 (3.3)	2 (3.3)	1.00
Dizziness	2 (6.7)	0 (0.0)	0.11
Rash	0 (0.0)	5(8.3)	0.17
Pruritus	2 (6.7)	1 (1.7)	0.26
Any adverse event*	5 (16.7)	10 (16.7)	1.00

* Patients experiencing adverse events during the course of treatment

In subgroups based on weight, no significant differences in the incidence of adverse events were observed. In patients weighing 20–39 kg, the incidence was 30.8% in the Paxlovid group vs. 23.8% in the control group (*P* = 0.7), and in patients weighing less than 20 kg, the incidence was 0.0% vs. 18.5%, respectively (*P* = 0.3). Additionally, no significant changes in laboratory parameters were observed (Supplementary Tables 3, 4). Supplementary Table 5 provides details of adverse events during the Paxlovid treatment period.

### Effectiveness of paxlovid

**Fig. 2:**
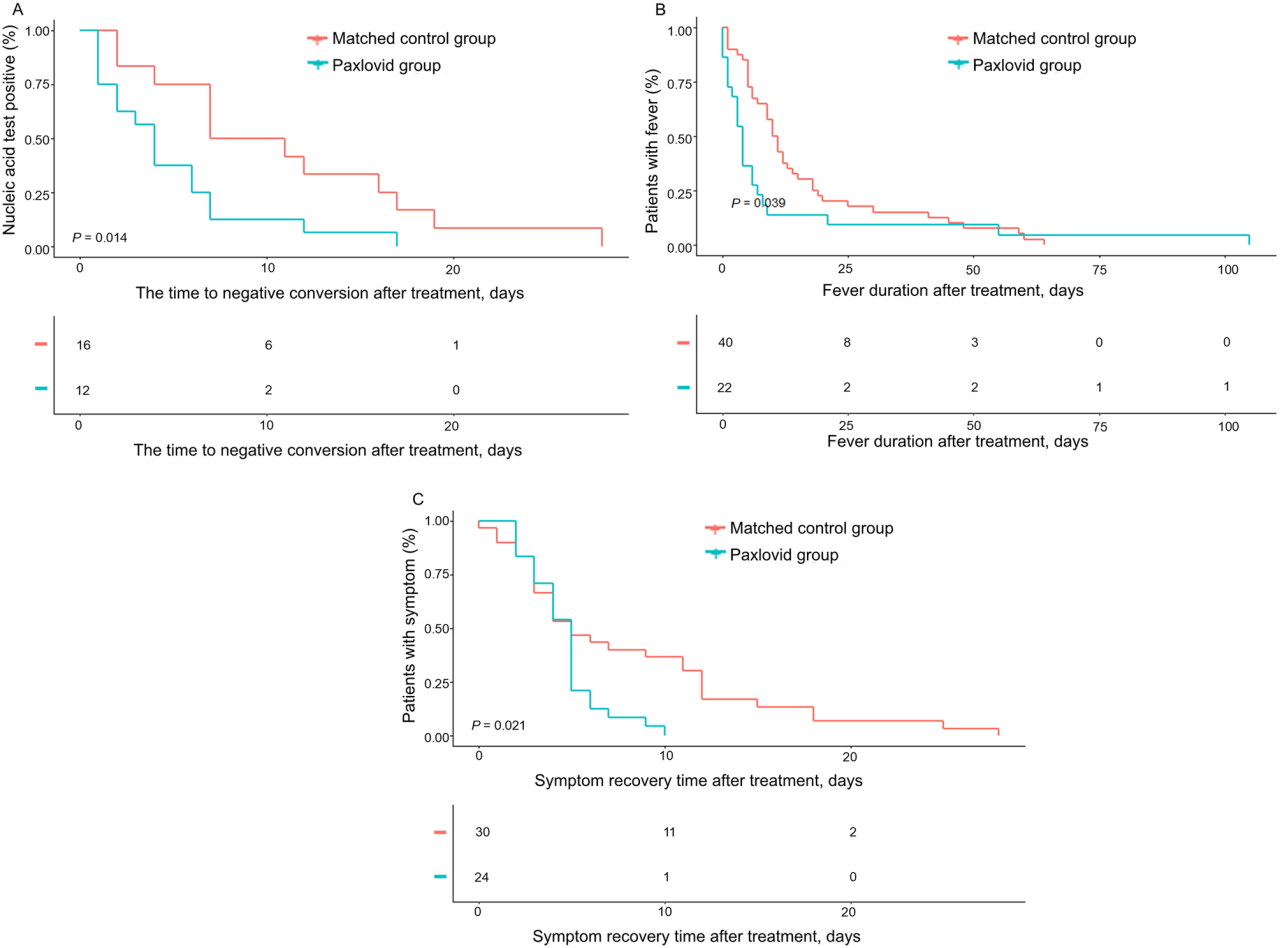
Kaplan–Meier Analysis of the Effects of Paxlovid Treatment. Efficacy outcome of (**A**) the time to negative conversion after treatment, (**B**) fever duration after treatment, and (**C**) symptom recovery time after treatment

Treatment with Paxlovid was associated with significantly shorter times to viral negative conversion, reduced fever duration, and faster symptom recovery compared to the control group. Specifically, the time to viral clearance was reduced from 11.0 days to 4.9 days (*P* = 0.01), fever duration decreased from 16.4 days to 11.2 days (*P* = 0.01), and symptom recovery was accelerated from 17.6 days to 4.6 days (*P* < 0.01) in the Paxlovid group (Fig. 2; Table 3). Among patients with underlying health conditions, Paxlovid treatment resulted in reductions of approximately 6 days for viral clearance, 4 days for fever duration, and 19 days for symptom recovery compared to the control group (Table 3). Univariate Cox regression analysis further indicated that treatment with Paxlovid was associated with a lower risk of prolonged symptom recovery (defined as recovery time exceeding five days), with an adjusted hazard ratio (aHR) of 0.4 (95% Confidence Interval [CI]: 0.1, 0.9) (Supplementary Table 6). In children under 12 years of age, symptom recovery was notably faster with Paxlovid, with recovery times reduced from 22.5 days to 5.1 days in children under 6 years, and from 13.8 days to 4.4 days in those aged 6–12 years (*P* < 0.01, *P* = 0.05) (Supplementary Table 1). Additionally, children with body weights of less than 20 kg and those weighing between 20 and 39 kg also demonstrated significant improvements in symptom recovery following Paxlovid treatment (*P* < 0.05) (Supplementary Table 2).

**Table 3:** Efficacy evaluations in the paxlovid group and matched control group stratified by underlying conditions

Variables, mean ± SD	Paxlovid group	Matched control group	*P*-value
**Total**	*n* = 30	*n* = 60	
The time to negative conversion after treatment, days	4.9 ± 4.4	11.0 ± 7.8	0.01
Fever duration after treatment, days	11.2 ± 24.0	16.4 ± 17.1	0.01
Symptom recovery time after treatment, days	4.6 ± 2.0	17.6 ± 25.5	< 0.01
Length of hospital stay, days	18.1 ± 19.8	18.4 ± 22.5	0.48
ICU admission before treatment, n (%)	11 (36.7)	3 (5.0)	< 0.01
ICU admission after treatment, n (%)	7 (23.3)	8 (13.3)	0.23
Transfer to ICU, n (%)	1 (3.3)	5 (8.3)	0.66
Death during hospitalization, n (%)	2 (6.7)	4 (6.7)	1.00
Death within 1 month of discharge, n (%)	4 (13.3)	5 (8.3)	0.47
Death within 6 months of discharge*, n (%)	4 (13.8)	7 (11.9)	0.82
**With underlying conditions**	*n* = 17	*n* = 34	
The time to negative conversion after treatment, days	5.6 ± 5.0	12.2 ± 8.6	0.05
Fever duration after treatment, days	17.3 ± 30.1	21.8 ± 20.4	0.06
Symptom recovery time after treatment, days	5.3 ± 2.3	24.6 ± 33.0	< 0.01
Length of hospital stay, days	24.8 ± 24.8	24.0 ± 28.4	0.72
ICU admission before treatment, n (%)	7 (41.2)	1 (2.9)	< 0.01
ICU admission after treatment, n (%)	6 (35.3)	6 (17.6)	0.16
Death during hospitalization, n (%)	2 (11.8)	2 (5.9)	0.59
Death within 1 month of discharge, n (%)	4 (23.5)	3 (8.8)	0.20
Death within 6 months of discharge^*^, n (%)	4 (25.0)	5 (15.2)	0.45
**Without underlying conditions**	*n* = 13	*n* = 26	
The time to negative conversion after treatment, days	3.2 ± 2.4	11.0	0.14
Fever duration after treatment, days	2.4 ± 2.7	7.3 ± 3.5	0.10
Symptom recovery time after treatment, days	3.9 ± 1.6	9.5 ± 5.6	< 0.01
Length of hospital stay, days	9.9 ± 4.2	11.0 ± 5.2	0.70
ICU admission before treatment, n (%)	4 (30.8)	2 (7.7)	0.15
ICU admission after treatment, n (%)	1 (7.7)	2 (7.7)	1.00
Death during hospitalization, n (%)	0 (0.0)	2 (7.7)	1.00
Death within 1 month of discharge, n (%)	0 (0.0)	2 (7.7)	0.54
Death within 6 months of discharge*, n (%)	0 (0.0)	2 (7.7)	0.54

* 1 person was lost to follow-up in each group

ICU, intensive care unit

**Fig. 3:**
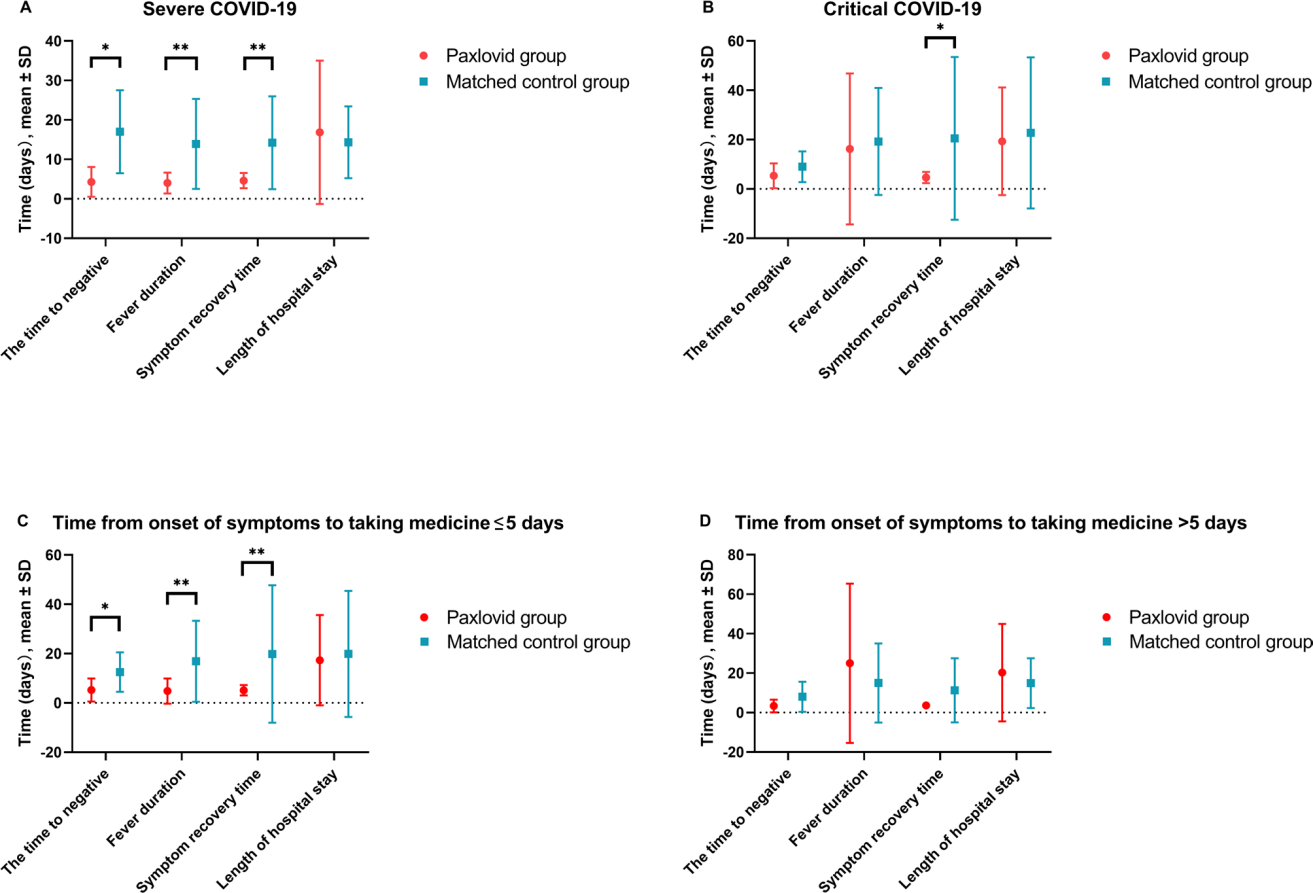
Efficacy Assessment of Paxlovid and Matched Control Groups, Stratified by Clinical Type and Timing of Medication Initiation. Efficacy outcomes of patients (**A**) with severe COVID-19, (**B**) with critical COVID-19, (**C**) who take medicine within five days from onset of symptoms and (**D**) who take medicine beyond five days from onset of symptoms. *P < 0.05, **P < 0.01

We categorized the participants based on the clinical severity of their COVID-19 and whether they received Paxlovid treatment within five days of symptom onset. In the group of patients with severe COVID-19, those treated with Paxlovid had significantly shorter times to viral clearance, fever resolution, and overall symptom recovery (Fig. 3). Among critically ill patients, the primary benefit of Paxlovid treatment was a reduction in symptom recovery time, with the treatment group recovering in 5.4 days compared to 21.2 days in the control group (*P* < 0.05) (Fig. 3B).

Regardless of whether treatment with Paxlovid was initiated beyond five days of symptom onset, patients in the Paxlovid group showed a shorter duration of symptom recovery and faster viral clearance compared to the control group. However, the most significant benefits were observed when Paxlovid was administered within five days of symptom onset. In this early treatment group, Paxlovid resulted in a substantially shorter time to viral clearance (5.2 vs. 12.5 days, *P* < 0.05), reduced fever duration (4.8 vs. 16.9 days, *P* < 0.01), and quicker symptom recovery (5.1 vs. 19.8 days, *P* < 0.01) compared to the control group (Fig. 3C).

### Pharmacokinetics

A total of 53 Nirmatrelvir concentrations from 30 patients were used to develop a pharmacokinetic (PK) model for Nirmatrelvir. The parameter estimates from the final model are presented in Supplementary Table 7. Diagnostic plots assessing the goodness-of-fit of the final model, shown in Supplementary Fig. 2, suggest it accurately predicts individual serum concentrations of Nirmatrelvir. Using the Bayesian feedback approach, the model allows for the estimation of individual steady-state plasma concentrations, which in turn enables the calculation of individual AUC_0 − 12 h_ values for Nirmatrelvir. Scatterplots comparing the AUC_0 − 12 h_ values between patients with severe and critical COVID-19 did not reveal significant differences (66.0 vs. 62.8 µg h/mL). However, in patients with underlying conditions, the AUC_0 − 12 h_ was slightly higher compared to those without underlying conditions, though this difference was not statistically significant (53.8 vs. 73.1 µg h/mL) (Supplementary Fig. 1A, B). Additionally, the Paxlovid group demonstrated a shorter time to viral clearance, reduced fever duration, and quicker symptom recovery, even in patients with Nirmatrelvir AUC_0 − 12 h_ values below 50 µg h/mL (*P* < 0.05) (Supplementary Fig. 1C).

## Discussion

Despite limited data on the safety and efficacy of Paxlovid for treating COVID-19 in children, our real-world study provides valuable insights. We found that among severely or critically ill children hospitalized with COVID-19, those treated with Paxlovid did not experience significant adverse effects. Furthermore, the time to clinical improvement and viral clearance was reduced by approximately 50% (5–6 days) compared to the control group, particularly in patients treated within 5 days of symptom onset. Notably, variations in Paxlovid blood levels, whether high or low, did not appear to impact treatment efficacy or exacerbate adverse effects.

Although this study did not investigate the subtypes of COVID-19, reports from the Chinese Center for Disease Control and Prevention indicate that the Omicron variant remains the predominant strain within the study population [36]. This observation aligns with findings in adults, where Paxlovid is both safe and effective under similar conditions. In adults, a meta-analysis of 23 studies involving 314,353 patients found no significant difference in the incidence of adverse events between the Paxlovid group and control group [15]. However, due to regulatory restrictions on Paxlovid use in children [37], few published studies are focusing on its safety in pediatric populations, especially those under 12 years old or with severe disease. A study involving five children treated with Paxlovid demonstrated promising results, but the sample size was small [8]. Additionally, a study of 31 critically ill pediatric patients found that Paxlovid was both effective and safe, but the study’s retrospective nature and the age differences between groups limit the generalizability of these findings [9].

The current study, which carefully recorded symptoms to differentiate between those induced by COVID-19 or Paxlovid, found that the incidence of adverse events was low and similar between the treatment and control groups. The most common adverse events were vomiting and diarrhea, suggesting that these symptoms were more likely related to the effects of COVID-19 itself, particularly during the Omicron variant prevalence, rather than to Paxlovid [38]. Dysgeusia, which is commonly reported as a side effect of Paxlovid in adults [15], was not observed in this study or other pediatric observational studies [3, 8]. This may be due to the difficulty children have in expressing symptoms, especially in critically ill patients who are intubated. Overall, the data suggests that Paxlovid is generally safe, with no significant adverse effects even in children < 6 years old or weighing < 20 kg.

Determining appropriate dosages for pediatric populations remains a challenge, particularly when the drug is not officially indicated for children. While the package insert recommends that Paxlovid tablets be taken whole, clinical pharmacology studies have shown no significant difference in pharmacokinetics between the suspension and the tablet formulations. Research on pediatric dosing is divided, with some studies recommending half the adult dosage, while others base the dosage on weight (e.g., body weight divided by 40 kg) [6–8]. Our study found that dosing on body weight, along with monitoring blood concentrations, did not appear to influence the efficacy of Paxlovid. It remained effective in younger children (< 6 years old or < 20 kg.), as evidenced by improvements in symptom resolution and time to viral clearance. Furthermore, the pharmacokinetic parameters observed in our study aligned with those reported in the adult (Supplementary Fig. 3) [39, 40], supporting the feasibility of treating critical COVID-19 in pediatric patients with Paxlovid.

While long COVID-19 has been reported in adults, characterized by symptoms such as fatigue, sleep disturbances and chronic cough [41], we did not observe any similar long-term symptoms in our pediatric cohort up to 6 months post-treatment. This suggests that the long-term prognosis of children seems to be less affected by COVID-19, although further studies with larger sample sizes and longer follow-up durations are needed to confirm these findings.

Adult studies have demonstrated that Paxlovid is effective in preventing the progression of COVID-19, reducing severity and lowering mortality rate [2, 15–19]. However, there is limited data on its efficacy in severe or critical pediatric cases. To our knowledge, this is the largest real-world study of Paxlovid in children with severe or critical COVID-19, with participants ranging from 2 months to over 12 years of age. Our findings suggest that the Paxlovid treatment was associated with a lower rate of ICU transfers and a shorter time to viral clearance compared to the control group. These results indicate that Paxlovid is effective in managing severe or critical cases of COVID-19, particularly in children with underlying conditions such as cancer. While no significant difference in mortality was observed between the two groups, this may be due to the more critical condition of the patients in the Paxlovid group. Nevertheless, the data suggests that early antiviral therapy with Paxlovid may reduce mortality in severe cases. Given these findings, early antiviral treatment with Paxlovid should be considered for children < 12 years old or weighing < 40 kg, particularly those with critical or underlying conditions, based on clinical need and parental consent.

This study has several limitations that should be considered. First, the two groups were not perfectly matched in terms of clinical severity. At the time of the study, there was limited high-quality evidence on the safety of Paxlovid for younger children, making it challenging to obtain consent from families of children with milder conditions. As a result, the Paxlovid group included patients with more severe illness. However, these patients did not experience a higher incidence of adverse events or mortality and showed a lower rate of ICU admissions compared to the control group, further suggesting that Paxlovid is both safe and effective. Second, this observational study may have been subject to some bias in symptom assessment, as children often cannot actively express symptoms. To mitigate this, we made careful efforts to document symptom changes in medical records and verified them with input from at least two pediatricians and nurses. This process aimed to reduce any potential bias in the data. Third, follow-up data were collected exclusively via telephone interviews. We ensured that each family was contacted for a minimum of 5 min during these calls. Additionally, our follow-up staff were thoroughly trained to conduct these interviews professionally and consistently.

In conclusion, this study supports the safety and effectiveness of Paxlovid for treating severe or critical COVID-19 in children, including those < 12 years of age or weighing < 40 kg, as well as children with underlying conditions. Based on these findings, we recommend considering Paxlovid for children with COVID-19, particularly when treatment is initiated within five days of symptom onset.

## Electronic supplementary material

Below is the link to the electronic supplementary material.

Supplementary Material 1

## Data Availability

No datasets were generated or analysed during the current study.

## Abbreviations

COVID-19: Coronavirus disease 2019
AUC_0 − 12h_: Area under the plasma concentration-­time curve from time 0 to 12 h
ICU: Intensive Care Unit
LC-MS/MS: Liquid chromatography-tandem mass spectrometry
AUC: Area under the curve
pSOFA: Pediatric sequential organ failure assessment
SIRS: Systemic inflammatory response syndrome
SD: Standard deviation
IQR: Interquartile range
aHR: Adjusted hazard ratio
CI: Confidence interval

## References

[1] WHO COVID-19 Dashboard. Geneva: World Health Organization. 2023 [Available from: https://covid19.who.int/October2023

[2] Hammond J, Leister-Tebbe H, Gardner A, et al. Oral nirmatrelvir for High-Risk, nonhospitalized adults with Covid-19. N Engl J Med. 2022;386(15):1397–408. 10.1056/NEJMoa2118542. 35172054 10.1056/NEJMoa2118542 PMC8908851

[3] Huang J, Yin D, Qin X, et al. Case report: application of nirmatrelvir/ritonavir to treat COVID-19 in a severe aplastic anemia child after allogeneic hematopoietic stem cell transplantation. Front Pead. 2022;10:935118. 10.3389/fped.2022.935118. 10.3389/fped.2022.935118 PMC9393292 36003491

[4] Vora SB, Englund JA, Trehan I, et al. Monoclonal antibody and antiviral therapy for Mild-to-Moderate COVID-19 in pediatric patients. Pediatr Infect Dis J. 2023;42(1):32–4. 10.1097/inf.0000000000003740. [published Online First: 2022/12/09]. 36476522 10.1097/INF.0000000000003740 PMC9725736

[5] Young C, Papiro T, Greenberg JH. Elevated tacrolimus levels after treatment with nirmatrelvir/ritonavir (Paxlovid) for COVID-19 infection in a child with a kidney transplant. Pediatr Nephrol. 2023;38(4):1387–88. 10.1007/s00467-022-05712-0. [published Online First: 2022/08/19]. 35982345 10.1007/s00467-022-05712-0 PMC9388350

[6] Shi S, Dong N, Ding Y, et al. [COVID-19 treated with oral Nirmatrelvir-Ritonavir in 3 children]. Zhonghua Er Ke Za Zhi. 2022;60(11):1168–71. 10.3760/cma.j.cn112140-20220701-00608. 36319152 10.3760/cma.j.cn112140-20220701-00608

[7] Li Y, Liu Y, Wen L, et al. Clinical efficacy analysis of paxlovid in children with hematological diseases infected with the Omicron SARS-CoV-2 new variant. Front Pediatr. 2023;11:1160929. 10.3389/fped.2023.1160929. [published Online First: 2023/05/14]. 37181421 10.3389/fped.2023.1160929 PMC10167044

[8] Yan G, Zhou J, Zhu H, et al. The feasibility, safety, and efficacy of paxlovid treatment in SARS-CoV-2-infected children aged 6–14 years: a cohort study. Annals Translational Med. 2022;10(11):619. 10.21037/atm-22-2791. 10.21037/atm-22-2791 PMC9263777 35813342

[9] Fan P, Ming M, Liu T, et al. Safety and efficacy of paxlovid in pediatric intensive care unit patients with COVID-19. Curr Med Chem. 2024. 10.2174/0109298673313885240621110518. 38956903 10.2174/0109298673313885240621110518

[10] Sun Y-K, Wang C, Lin P-Q, et al. Severe pediatric COVID-19: a review from the clinical and Immunopathophysiological perspectives. World J Pediatr. 2024. 10.1007/s12519-023-00790-y. 38321331 10.1007/s12519-023-00790-y PMC11052880

[11] Wang Z, Zhao S, Tang Y, et al. Potentially effective drugs for the treatment of COVID-19 or MIS-C in children: a systematic review. Eur J Pediatr. 2022;181(5):2135–46. 10.1007/s00431-022-04388-w. [published Online First: 2022/02/23]. 35192051 10.1007/s00431-022-04388-w PMC8861482

[12] Chiotos K, Hayes M, Kimberlin DW, et al. Multicenter interim guidance on use of antivirals for children with coronavirus disease 2019/severe acute respiratory syndrome coronavirus 2. J Pediatr Infect Dis Soc. 2021;10(1):34–48. 10.1093/jpids/piaa115. [published Online First: 2020/09/13]. 10.1093/jpids/piaa115 PMC7543452 32918548

[13] Campbell JI, Ocwieja KE, Nakamura MM. A call for pediatric COVID-19 clinical trials. Pediatrics. 2020;146(2). 10.1542/peds.2020-1081. [published Online First: 2020/05/14]. 10.1542/peds.2020-1081 32398330

[14] China NHCotPsRo. Diagnosis and treatment plan for COVID-19 (trial version 10). China Med. 2023;18:161–66. 10.3760/cma.j.issn.1674-2397.2023.01.001.

[15] Amani B, Amani B. Efficacy and safety of nirmatrelvir/ritonavir (Paxlovid) for COVID-19: A rapid review and meta-analysis. J Med Virol. 2023;95(2):e28441. 10.1002/jmv.28441. 36576379 10.1002/jmv.28441 PMC9880713

[16] Anwar K, Nguyen L, Nagasaka M, Ou S-HI, Chan A. Overview of Drug-Drug interactions between Ritonavir-Boosted nirmatrelvir (Paxlovid) and targeted therapy and supportive care for lung Cancer. JTO Clin Res Rep. 2023;4(2):100452. 10.1016/j.jtocrr.2022.100452. 36568522 10.1016/j.jtocrr.2022.100452 PMC9759297

[17] Devresse A, Sébastien B, De Greef J, et al. Safety, efficacy, and relapse of Nirmatrelvir-Ritonavir in kidney transplant recipients infected with SARS-CoV-2. Kidney Int Rep. 2022;7(11):2356–63. 10.1016/j.ekir.2022.08.026. 36060621 10.1016/j.ekir.2022.08.026 PMC9420244

[18] Malden DE, Hong V, Lewin BJ, et al. Hospitalization and emergency department encounters for COVID-19 after paxlovid Treatment - California, December 2021-May 2022. MMWR Morbidity Mortal Wkly Rep. 2022;71(25):830–33. 10.15585/mmwr.mm7125e2. 10.15585/mmwr.mm7125e2 35737591

[19] Najjar-Debbiny R, Gronich N, Weber G, et al. Effectiveness of paxlovid in reducing severe coronavirus disease 2019 and mortality in High-Risk patients. Clin Infect Diseases: Official Publication Infect Dis Soc Am. 2023;76(3):e342–49. 10.1093/cid/ciac443. 10.1093/cid/ciac443 PMC9214014 35653428

[20] Beijnen EMS, Odumade OA, Haren SDV. Molecular determinants of the early life immune response to COVID-19 infection and immunization. Vaccines (Basel). 2023;11(3). 10.3390/vaccines11030509. [published Online First: 2023/03/31]. 10.3390/vaccines11030509 PMC10052886 36992093

[21] Liguoro I, Pilotto C, Bonanni M, et al. SARS-COV-2 infection in children and newborns: a systematic review. Eur J Pediatr. 2020;179(7):1029–46. 10.1007/s00431-020-03684-7. [published Online First: 2020/05/20]. 32424745 10.1007/s00431-020-03684-7 PMC7234446

[22] Cui X, Zhao Z, Zhang T, et al. A systematic review and meta-analysis of children with coronavirus disease 2019 (COVID-19). J Med Virol. 2021;93(2):1057–69. 10.1002/jmv.26398. [published Online First: 2020/08/08]. 32761898 10.1002/jmv.26398 PMC7436402

[23] Irfan O, Muttalib F, Tang K, Jiang L, Lassi ZS, Bhutta Z. Clinical characteristics, treatment and outcomes of paediatric COVID-19: a systematic review and meta-analysis. Arch Dis Child. 2021;106(5):440–8. 10.1136/archdischild-2020-321385. [published Online First: 2021/02/18]. 33593743 10.1136/archdischild-2020-321385 PMC8070630

[24] Stokes EK, Zambrano LD, Anderson KN, et al. Coronavirus disease 2019 case Surveillance - United states, January 22-May 30, 2020. MMWR Morb Mortal Wkly Rep. 2020;69(24):759–65. 10.15585/mmwr.mm6924e2. [published Online First: 2020/06/20]. 32555134 10.15585/mmwr.mm6924e2 PMC7302472

[25] Kim L, Whitaker M, O’Halloran A, et al. Hospitalization rates and characteristics of children aged < 18 years hospitalized with Laboratory-Confirmed COVID-19 - COVID-NET, 14 states, March 1-July 25, 2020. MMWR Morb Mortal Wkly Rep. 2020;69(32):1081–88. 10.15585/mmwr.mm6932e3. [published Online First: 2020/08/14]. 32790664 10.15585/mmwr.mm6932e3 PMC7440125

[26] Götzinger F, Santiago-García B, Noguera-Julián A, et al. COVID-19 in children and adolescents in europe: a multinational, multicentre cohort study. Lancet Child Adolesc Health. 2020;4(9):653–61. 10.1016/s2352-4642(20)30177-2. [published Online First: 2020/07/01]. 32593339 10.1016/S2352-4642(20)30177-2 PMC7316447

[27] Shekerdemian LS, Mahmood NR, Wolfe KK, et al. Characteristics and outcomes of children with coronavirus disease 2019 (COVID-19) infection admitted to US and Canadian pediatric intensive care units. JAMA Pediatr. 2020;174(9):868–73. 10.1001/jamapediatrics.2020.1948. [published Online First: 2020/05/12]. 32392288 10.1001/jamapediatrics.2020.1948 PMC7489842

[28] Mukkada S, Bhakta N, Chantada GL, et al. Global characteristics and outcomes of SARS-CoV-2 infection in children and adolescents with cancer (GRCCC): a cohort study. Lancet Oncol. 2021;22(10):1416–26. 10.1016/s1470-2045(21)00454-x. [published Online First: 2021/08/30]. 34454651 10.1016/S1470-2045(21)00454-X PMC8389979

[29] Steliarova-Foucher E, Colombet M, Ries LAG, et al. International incidence of childhood cancer, 2001-10: a population-based registry study. Lancet Oncol. 2017;18(6):719–31. 10.1016/S1470-2045(17)30186-9. 28410997 10.1016/S1470-2045(17)30186-9 PMC5461370

[30] WHO. CureAll framework: WHO global initiative for childhood cancer: increasing access, advancing quality, saving lives. 2021 [Available from: https://apps.who.int/iris/handle/10665/347370

[31] McWilliam SJ, Hawcutt DB. Solving the problem of dose optimization of children’s medicines. Expert Rev Clin Pharmacol. 2018;11(3):205–08. 10.1080/17512433.2018.1431528. 29355041 10.1080/17512433.2018.1431528

[32] Matalová P, Urbánek K, Anzenbacher P. Specific features of pharmacokinetics in children. Drug Metab Rev. 2016;48(1):70–9. 10.3109/03602532.2015.1135941. 26828377 10.3109/03602532.2015.1135941

[33] Matics TJ, Sanchez-Pinto LN. Adaptation and validation of a pediatric sequential organ failure assessment score and evaluation of the Sepsis-3 definitions in critically ill children. JAMA Pediatr. 2017;171(10):e172352. 10.1001/jamapediatrics.2017.2352. 28783810 10.1001/jamapediatrics.2017.2352 PMC6583375

[34] Singer M, Deutschman CS, Seymour CW, et al. The third international consensus definitions for Sepsis and septic shock (Sepsis-3). JAMA. 2016;315(8):801–10. 10.1001/jama.2016.0287. 26903338 10.1001/jama.2016.0287 PMC4968574

[35] Jing W, Yuanjie Z, Xiaoxia D, Nan S, Wei W. Clinical characteristics of the SARS-CoV-2 Omicron variant infection in children with different basic diseases. Chin Pediatr Emerg Med. 2022;29(10):779–83. 10.3760/cma.j.issn.1673-4912.2022.10.005.

[36] CDC. The situation of the new coronavirus infection in the country [in Chinese] 2023 [Available from: https://www.chinacdc.cn/jkzt/crb/zl/szkb_11803/jszl_13141/202302/t20230225_263964.html accessed 2023-02-25.

[37] Lamb YN. Nirmatrelvir plus ritonavir: first approval. Drugs. 2022;82(5):585–91. 10.1007/s40265-022-01692-5. 10.1007/s40265-022-01692-5 PMC8933659 35305258

[38] Jiao FY, Ma L. [Strengthening the prevention and treatment of Omicron infection in children]. Zhongguo Dang Dai Er Ke Za Zhi. 2022;24(4):345–49. 10.7499/j.issn.1008-8830.2201001. [published Online First: 2022/05/10]. 35527405 10.7499/j.issn.1008-8830.2201001 PMC9044981

[39] Liu C, Zhu M, Cao L, et al. Simultaneous determination of nirmatrelvir and Ritonavir in human plasma using LC-MS/MS and its Pharmacokinetic application in healthy Chinese volunteers. Biomed Chromatogr. 2022;36(11):e5456. 10.1002/bmc.5456. 35881032 10.1002/bmc.5456

[40] Toussi SS, Neutel JM, Navarro J, et al. Pharmacokinetics of oral nirmatrelvir/ritonavir, a protease inhibitor for treatment of COVID-19, in subjects with renal impairment. Clin Pharmacol Ther. 2022;112(4):892–900. 10.1002/cpt.2688. 35712797 10.1002/cpt.2688 PMC9349773

[41] Desai AD, Lavelle M, Boursiquot BC, Wan EY. Long-term complications of COVID-19. Am J Physiol Cell Physiol. 2022;322(1). 10.1152/ajpcell.00375.2021. 10.1152/ajpcell.00375.2021 PMC8721906 34817268

