# Supplementary material for: Paxlovid for the treatment of severe or critical COVID-19 in children
Source: BMC Pediatr. 2025 Jul 2;25:493. doi: 10.1186/s12887-025-05807-1 (PMC12220247; doi:10.1186/s12887-025-05807-1)
Supplement: Supplementary file 1 — Supplementary Material 1 [file 12887_2025_5807_MOESM1_ESM.docx]

**Supplementary Material**

**Table and figure of contents**

Appendix S1 The SARS-CoV-2 nucleic acid test.

Appendix S2 Sampling and detection of Paxlovid in plasma.

Appendix S3 COVID-19 clinical types.

Appendix S4 Statistics about pharmacokinetics analysis.

Appendix S5 The criteria for the usage of Paxlovid.

Table S1 Efficacy and safety outcomes stratified by onset ages of COVID-19 (year).

Table S2 Efficacy and safety outcomes stratified by weight.

Table S3 Laboratory parameters before therapy in two groups.

Table S4 Laboratory parameters after therapy (5 ± 2 days) in two groups.

Table S5 Adverse events of Paxlovid in five children with COVID-19.

Table S6 Effect of Paxlovid on outcomes after treatment.

Table S7 Population pharmacokinetic parameter estimates from the Nirmatrelvir model.

Figure S1 Scatterplots for Nirmatrelvir AUC_0-12h_ and efficacy outcomes.

Figure S2 Diagnostic goodness-of-fit plots of the Nirmatrelvir model.

Figure S3 Serum concentration-time profiles of Nirmatrelvir.

**Appendix S1 The SARS-CoV-2 nucleic acid test.**

The SARSCoV-2 viral load was detected and quantified by reverse transcription-polymerase chain reaction kit (Guangzhou Daan Gene, China and Hunan Sansure Biotech, China) using nasopharyngeal swabs during admission. A cycle threshold (Ct) value >40 of PCR test for both ORF1ab and N gene was considered negative. For antigen detection, throat swabs were collected and detected using a commercial colloid gold assay kit (Nanjing Vazyme, China) targeting the SARS-CoV-2 N antigen.

**Appendix S2 Sampling and detection of Paxlovid in plasma.**

An opportunistic sampling strategy of plasma was performed. The precise time of paxlovid administration and sampling were able to be indexed. One to two milliliters of peripheral blood were collected each time. Plasma samples were separated by centrifugation for 5 minutes at 15,000 rpm immediately after sampling. Specimens were transported in a specimen box with a lid and an ice pack to the central laboratory of Zhejiang Wenzhou Medical University. The concentration quantification was performed using a validated high-performance liquid chromatography-tandem mass spectrometry (LC-MS/MS) assay. The mass spectrometer was operated in the positive ion mode and monitored the transition ions m/z 500.2→110.0 and 721.3→296.1 for nirmatrelvir and ritonavir, respectively. The intra- and inter-day assay coefficients of variation were less than 10%. The lower limit for quantification was 1 ng/mL.

**Appendix S3 COVID-19 clinical types.**

1. Mild: Upper respiratory tract infections are the main manifestations, such as dry throat, sore throat, cough, fever, etc

2. Moderate: Persistent high fever for > 3 days or/(and) cough, shortness of breath, etc., but respiratory rate (RR) < 30 bpm, oxygen saturation > 93% when inhaling air at rest. Characteristic findings of COVID-19 pneumonia can be seen on imaging.

3. Severe: Comply with any of the following: 1) Ultra-high fever or persistent high fever for more than 3 days; 2) Shortness of breath (< 2 months of age, RR ≥ 60 beats/min; 2~12 months old, RR ≥ 50 beats/min; 1~5 years old, RR ≥ 40 times/min; > 5 years of age, RR ≥ 30 beats/minute), excluding the effects of fever and crying; 3) In the resting state, the oxygen saturation is ≤ 93% when inhaling air; 4) Nasal flaring, retraction, stridor or wheezing; 5) Disturbance of consciousness or convulsions; 6) Refusal to eat or feeding difficulties, with signs of dehydration.

4. Critical: Comply with any of the following: 1) Respiratory failure and mechanical ventilation; 2) Shock; 3) Combined with other organ failure requiring ICU monitoring and treatment.

**Appendix S4 Statistics about pharmacokinetics analysis.**

Nonlinear mixed-effects modeling (NONMEM) program (version 7.4, Icon Development Solutions, Ellicott City, MD, United States) and Pirana (version 2.9.7) was used to perform PK analysis of nirmatrelvir/ritonavir. R (Version 3.6.1) was used to analyze the NONMEM output. The first-order conditional estimation with the interaction between inter-patient variability and residual variability was used for model development.

**Appendix S5 The criteria for the usage of Paxlovid.**

**INDICATIONS AND USAGE**

PAXLOVID which includes nirmatrelvir, a severe acute respiratory syndrome coronavirus 2 (SARS-CoV-2) main protease (M^pro^: also referred to as 3CL^pro^ or nsp5 protease) inhibitor, and ritonavir, an HIV-1 protease inhibitor and CYP3A inhibitor, is indicated for the treatment of mild-to-moderate coronavirus disease 2019 (COVID-19) in adults who are at high risk for progression to severe COVID-19, including hospitalization or death.

Limitations of Use

PAXLOVID is not approved for use as pre-exposure or post-exposure prophylaxis for prevention of COVID-19.

**DOSAGE AND ADMINISTRATION**

PAXLOVID is nirmatrelvir tablets co-packaged with ritonavir tablets.

Nirmatrelvir must be co-administered with ritonavir.

•Initiate PAXLOVID treatment as soon as possible after diagnosis of COVID-19 and within 5 days of symptom onset.

•Administer orally with or without food.

•Dosage: 300 mg nirmatrelvir (two 150 mg tablets) with 100 mg ritonavir (one 100 mg tablet), with all 3 tablets taken together twice daily for 5 days.

•Dose reduction for moderate renal impairment (eGFR ≥30 to <60 mL/min): 150 mg nirmatrelvir (one 150 mg tablet) with 100 mg ritonavir (one 100 mg tablet), with both tablets taken together twice daily for 5 days.

•PAXLOVID is not recommended in patients with severe renal impairment (eGFR <30 mL/min).

•PAXLOVID is not recommend in patients with severe hepatic impairment (Child-Pugh Class C).

**DOSAGE FORMS AND STRENGTHS**

•Tablets: nirmatrelvir 150 mg

•Tablets: ritonavir 100 mg

**CONTRAINDICATIONS**

•History of clinically significant hypersensitivity reactions to the active ingredients (nirmatrelvir or ritonavir) or any other components.

•Co-administration with drugs highly dependent on CYP3A for clearance and for which elevated concentrations are associated with serious and/or life-threatening reactions.

•Co-administration with potent CYP3A inducers where significantly reduced nirmatrelvir or ritonavir plasma concentrations may be associated with the potential for loss of virologic response and possible resistance.

**WARNINGS AND PRECAUTIONS**

•The concomitant use of PAXLOVID and certain other drugs may result in potentially significant drug interactions. Consult the Full Prescribing Information prior to and during treatment for potential drug interactions.

•Hypersensitivity Reactions: Anaphylaxis, serious skin reactions (including toxic epidermal necrolysis and Stevens-Johnson syndrome), and other hypersensitivity reactions have been reported with PAXLOVID. If signs and symptoms of a clinically significant hypersensitivity reaction or anaphylaxis occur, immediately discontinue PAXLOVID and initiate appropriate medications and/or supportive care.

•Hepatotoxicity: Hepatic transaminase elevations, clinical hepatitis, and jaundice have occurred in patients receiving ritonavir.

•HIV-1 Drug Resistance: PAXLOVID use may lead to a risk of HIV-1 developing resistance to HIV protease inhibitors in individuals with uncontrolled or undiagnosed HIV-1 infection.

**ADVERSE REACTIONS**

Most common adverse reactions (incidence ≥1% and greater incidence than in the placebo group) are dysgeusia and diarrhea.

**To report SUSPECTED ADVERSE REACTIONS, contact Pfizer Inc. at 1-800-438-1985 or FDA at 1-800-FDA-1088 or www.fda.gov/medwatch.**

**DRUG INTERACTIONS**

Co-administration of PAXLOVID can alter the plasma concentrations of other drugs and other drugs may alter the plasma concentrations of PAXLOVID. Consider the potential for drug interactions prior to and during PAXLOVID therapy and review concomitant medications during PAXLOVID therapy.

**Table S1 Efficacy and safety outcomes stratified by onset ages of COVID-19 (year).**

| **Variables, mean ± SD** | **Paxlovid group (n = 30)** | **Matched control group (n = 60)** | ***P-*value** |
| --- | --- | --- | --- |
| **Age <6 years old** | n = 10 | n = 23 |  |
| The time to negative conversion after treatment, days | 7.0 ±5.6 | 6.0 ± 3.9 | 0.92 |
| Fever duration after treatment, days | 5.9 ± 7.1 | 18.6 ± 19.3 | <0.05 |
| Symptom recovery time after treatment, days | 5.1 ± 2.7 | 22.5 ± 35.3 | <0.01 |
| Length of hospital stay, days | 21.8 ± 17.3 | 24.4 ± 32.1 | 0.79 |
| Patients with adverse events, n (%) | 0 (0.0) | 4 (17.4) | 0.29 |
|  |  |  |  |
| **Age = 6-11 years old** | n = 17 | n = 25 |  |
| The time to negative conversion after treatment, days | 3.2 ± 2.4 | 10.2 ± 5.8 | <0.05 |
| Fever duration after treatment, days | 15.1 ± 30.7 | 14.8 ± 13.4 | 0.07 |
| Symptom recovery time after treatment, days | 4.4 ± 1.9 | 13.8 ± 13.7 | <0.05 |
| Length of hospital stay, days | 16.4 ± 23.1 | 14.0 ± 10.7 | 0.19 |
| Patients with adverse events, n (%) | 4 (23.5) | 6 (24.0) | 0.97 |
|  |  |  |  |
| **Age ≥12 years old** | n = 3 | n = 12 |  |
| The time to negative conversion after treatment, days | - | 23.5 ± 6.4 | - |
| Fever duration after treatment, days | 5.0 ± 5.7 | 15.0 ± 20.7 | 0.55 |
| Symptom recovery time after treatment, days | 4.3 ± 0.6 | 14.6 ± 17.8 | 0.19 |
| Length of hospital stay, days | 15.0 ± 6.6 | 15.2 ± 13.8 | 0.43 |
| Patients with adverse events, n (%) | 1(33.3) | 0 (0.0) | 0.20 |

**Table S2 Efficacy and safety outcomes stratified by weight.**

| **Variables, mean ± SD** | **Paxlovid group (n = 30)** | **Matched control group (n = 60)** | ***P-*value** |
| --- | --- | --- | --- |
| **Weight <20 kg** | n = 11 | n = 27 |  |
| The time to negative conversion after treatment, days | 4.2 ± 3.8 | 7.2 ± 4.3 | 0.18 |
| Fever duration after treatment, days | 16.5 ± 35.9 | 19.0 ± 19.7 | <0.05 |
| Symptom recovery time after treatment, days | 4.4 ± 1.6 | 22.1 ± 33.9 | <0.01 |
| Length of hospital stay, days | 20.1 ± 17.7 | 24.6 ± 30.8 | 0.72 |
| Patients with adverse events, n (%) | 0 (0.0) | 5 (18.5) | 0.30 |
|  |  |  |  |
| **Weight =20-39 kg** | n = 13 | n = 21 |  |
| The time to negative conversion after treatment, days | 5.6 ± 6.5 | 13.7 ± 8.9 | 0.09 |
| Fever duration after treatment, days | 5.4 ± 6.2 | 16.1 ± 15.6 | <0.05 |
| Symptom recovery time after treatment, days | 5.2 ± 2.4 | 15.7 ± 15.6 | <0.05 |
| Length of hospital stay, days | 14.6 ± 17.8 | 15.2 ± 12.0 | 0.24 |
| Patients with adverse events, n (%) | 4 (30.8) | 5 (23.8) | 0.70 |
|  |  |  |  |
| **Weight ≥40 kg** | n = 6 | n = 12 |  |
| The time to negative conversion after treatment, days | 5.3 ± 2.9 | - | - |
| Fever duration after treatment, days | 13.2 ± 23.6 | 8.0 ± 7.3 | 0.52 |
| Symptom recovery time after treatment, days | 3.6 ± 1.1 | 9.2 ± 7.1 | 0.08 |
| Length of hospital stay, days | 22.5 ± 28.7 | 10.1 ± 4.5 | 0.39 |
| Patients with adverse events, n (%) | 1 (16.7) | 0 (0.0) | 0.33 |

**Table S3** **Laboratory parameters before therapy in two groups.**

| **Variables, mean ± SD** | **Before therapy** | | ***P-*value** |
| --- | --- | --- | --- |
|  | **Paxlovid group (n=30)** | **Matched control group (n=60)** |  |
| **Blood routine** |  |  |  |
| White blood cell count, ×10^9^/L | 7.6 ± 5.6 | 9.2 ± 6.6 | 0.25 |
| Lymphocyte, % | 22.2 ± 16.6 | 25.8 ± 19.6 | 0.35 |
| Hemoglobin (g/L) | 109.8 ± 23.5 | 114.5 ± 23.8 | 0.34 |
| Platelet count, ×10^9^/L | 238.4 ± 134.9 | 245.2 ± 147.7 | 0.98 |
| **Blood biochemistry** |  |  |  |
| Total protein, g/L | 65.1 ± 9.0 | 72.0 ± 61.4 | 0.41 |
| Albumin, g/L | 39.3 ± 6.0 | 37.6 ± 5.3 | 0.07 |
| Lactate dehydrogenase, U/L | 502.2 ± 530.4 | 526.7 ± 478.1 | 0.78 |
| ALT, U/L | 41.5 ± 88.0 | 75.4 ± 137.3 | 0.31 |
| AST, U/L | 74.9 ± 164.0 | 128.8 ± 269.0 | 0.16 |
| Serum creatinine, μmol/L | 31.3 ± 13.7 | 33.7 ± 20.8 | 0.87 |
| eGFR, mL/min/1.73m^2^ | 236.3 ± 240.2 | 215.2 ± 150.2 | 0.91 |
| Urea, mmol/L | 9.9 ± 27.6 | 4.6 ± 2.3 | 0.84 |
| Total bilirubin, μmol/L | 9.1 ± 4.1 | 11.1 ± 24.5 | 0.02 |
| Direct bilirubin, μmol/L | 1.9 ± 0.8 | 3.9 ± 13.1 | 0.51 |
| C-reactive protein, mg/L | 35.6 ± 53.1 | 27.2 ± 38.1 | 1.00 |
| **Coagulation function** |  |  |  |
| Prothrombin time, s | 12.7 ± 2.3 | 12.8 ± 2.5 | 0.79 |
| APTT, s | 31.7 ± 9.3 | 32.0 ± 7.3 | 0.60 |
| Fibrinogen, g/L | 3.6 ± 1.8 | 3.2 ± 1.3 | 0.48 |
| D-dimer, ng/mL | 2.7 ± 6.1 | 1.8 ± 5.2 | 0.26 |

ALT, alanine aminotransferase; AST, aspartate aminotransferase; eGFR, estimated glomerular filtration rate; APTT, activated partial thromboplastin clotting time.

**Table** **S4 Laboratory parameters after therapy (5 ± 2 days) in two groups.**

| **Variables, mean ± SD** | **After therapy (5 ± 2 days)** | | ***P-*value** |
| --- | --- | --- | --- |
|  | **Paxlovid group (n=30)** | **Matched control group (n=60)** |  |
| **Blood routine** |  |  |  |
| White blood cell count, ×10^9^/L | 8.9 ± 5.4 | 9.2 ± 5.7 | 0.69 |
| Lymphocyte, % | 20.1 ± 15.4 | 30.5 ± 21.9 | 0.03 |
| Hemoglobin (g/L) | 107.8 ± 23.5 | 108.3 ± 23.5 | 0.69 |
| Platelet count, ×10^9^/L | 296.2 ± 189.4 | 284.1 ± 190.4 | 0.74 |
| **Blood biochemistry** |  |  |  |
| Total protein, g/L | 66.7 ± 7.4 | 68.3 ± 9.6 | 0.72 |
| Albumin, g/L | 36.0 ± 5.0 | 37.1 ± 5.0 | 0.65 |
| Lactate dehydrogenase, U/L | 537.7 ± 844.4 | 436.7 ± 316.8 | 0.79 |
| ALT, U/L | 23.8 ± 22.3 | 56.5 ± 57.3 | <0.01 |
| AST, U/L | 68.9 ± 131.0 | 53.2 ± 48.5 | 0.43 |
| Serum creatinine, μmol/L | 27.7 ± 13.1 | 27.4 ± 10.8 | 0.85 |
| eGFR, mL/min/1.73m^2^ | 217.8 ± 68.6 | 231.7 ± 153.7 | 0.76 |
| Urea, mmol/L | 4.8 ± 1.8 | 4.4 ± 2.2 | 0.15 |
| Total bilirubin, μmol/L | 9.3 ± 5.4 | 11.6 ± 18.1 | 0.24 |
| Direct bilirubin, μmol/L | 2.3 ± 3.0 | 3.7 ± 9.2 | 0.72 |
| C-reactive protein, mg/L | 15.1 ± 30.7 | 15.7 ± 28.7 | 0.05 |
| **Coagulation function** |  |  |  |
| Prothrombin time, s | 11.5 ± 1.8 | 11.4 ± 1.0 | 0.95 |
| APTT, s | 29.2 ± 7.5 | 27.5 ± 5.0 | 0.64 |
| Fibrinogen, g/L | 2.5 ± 1.4 | 2.9 ± 1.2 | 0.19 |
| D-dimer, ng/mL | 1.7 ± 2.8 | 2.5 ± 4.3 | 0.56 |

ALT, alanine aminotransferase; AST, aspartate aminotransferase; eGFR, estimated glomerular filtration rate; APTT, activated partial thromboplastin clotting time.

**Table S5** **Adverse events of Paxlovid in five children with COVID-19.**

| **Variables** | **Case 1** | **Case 2** | **Case 3** | **Case 4** | **Case 5** |
| --- | --- | --- | --- | --- | --- |
| Gender | Male | Male | Female | Male | Female |
| Age, year | 8 | 16 | 7 | 12 | 11 |
| BMI, kg/m^2^ | 17.79 | 18.97 | 14.98 | 11.96 | 20.85 |
| Underlying conditions | - | Intracranial malignancy | - | leukemia | Juvenile idiopathic arthritis |
| Antibiotics | Azithromycin, Amoxicillin | Sulbactam and Cefoperazone, Vancomycin, Meropenem, tigecycline | Linezolid, Sulbactam and Cefoperazone | Sulfamethoxazole | Sulfamethoxazole, Linezolid |
| Other antiviral drugs | - | - | - | - | - |
| Combination medications* | - |  | - | - | Cyclosporine |
| Nirmatrelvir/ ritonavir, mg | 225/75 q12h, 5 days | 150/100 q12h, 5 days | 150/100 q12h, 5 days | 150/100 q12h, 5 days | 225/75 q12h, 5days |
| Adverse events | Pruritus | Pruritus | Dizziness, | Vomiting, dizziness | Diarrhea |

* Medications on the Paxlovid label with known drug interactions

**Table S6 Effect of Paxlovid on outcomes after treatment.**

| **Variables** | **Matched control group** | **Paxlovid group** | | | |
| --- | --- | --- | --- | --- | --- |
|  |  | **Case (%)** | **HR (CI)** | **aHR* (CI)** | **aHR** (CI)** |
| The time to negative conversion after treatment >5 days | 1.0 (ref) | 6 (37.5) | 0.8 (0.3, 2.5) | 0.5 (0.1, 2.3) | 0.9 (0.3, 3.0) |
| Fever duration after treatment >10 days | 1.0 (ref) | 3 (13.6) | 0.5 (0.1, 1.7) | 0.5 (0.1, 1.8) | 0.5 (0.1, 1.7) |
| Symptom recovery time after treatment >5 days | 1.0 (ref) | 5 (20.8) | 0.3 (0.1, 0.8) | 0.4 (0.1, 0.9) | 0.4 (0.1, 1.0) |
| Transfer to ICU ^a^ | 1.0 (ref) | 1 (3.3) | 0.7 (0.1, 5.7) | 0.7 (0.1, 6.0) | 1.0 (0.1, 9.6) |
| Death within 6 months | 1.0 (ref) | 4 (13.3) | 1.5 (0.9, 2.4) | - | 1.5 (0.9, 2.5) |

* Adjust by age and sex.

** Adjust by underlying conditions.

^a^ Period of hospitalization.

ICU, intensive care unit; HR, hazard ratio; CI, confidence interval.

**Table S7** **Population pharmacokinetic parameter estimates from the Nirmatrelvir model.**

| **Parameter** | **Estimate** | **Relative Standard** | **Shrinkage** |
| --- | --- | --- | --- |
| Fixed Effects |  |  |  |
| F | 0.518 | 29% |  |
| Ka | 0.243 | 36% |  |
| CL [L/h] | 1.33 | 28% |  |
| V [L] | 8.03 | 19% |  |
| Between-subject Variability (BSV^a^) |  |  |  |
| BSV_CL [%CV] | 57.9% | 25% | [18%] |
| Residual Variability (RUV) |  |  |  |
| Proportional Error [%CV] | 68.70% | 32% | [9%] |
| ^a^ BSV calculated as $\sqrt{e^{\omega^{2}}-1}$  F, bioavailability; Ka, estimation of absorption rate constant; CL, renal clearance. | | | |


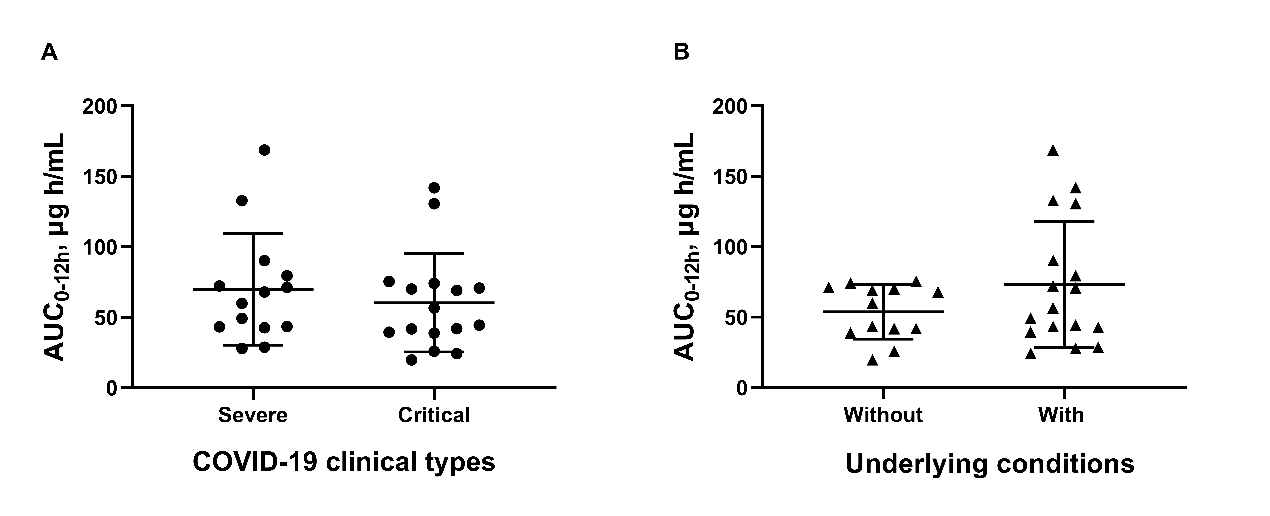


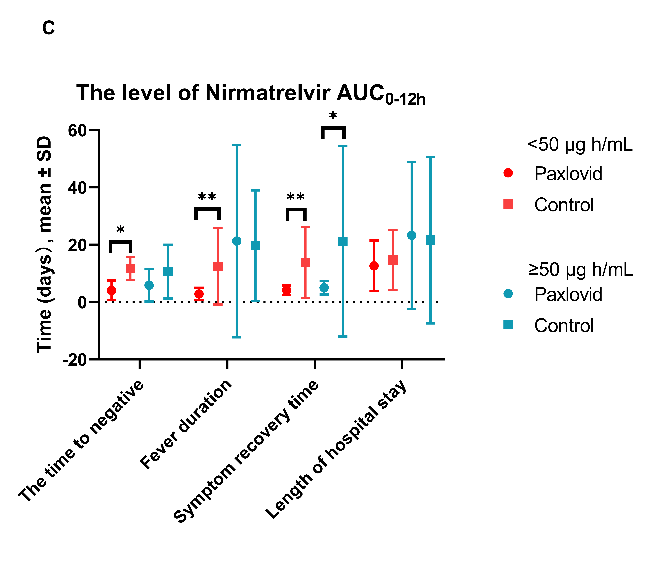


**Figure S1** Scatterplots for Nirmatrelvir AUC_0-12h_ (A) in different COVID-19 clinical types; (B) in whether with underlying conditions; And (C) efficacy outcomes stratified by the level of Nirmatrelvir AUC_0-12h_. **P* < 0.05, ***P* < 0.01.


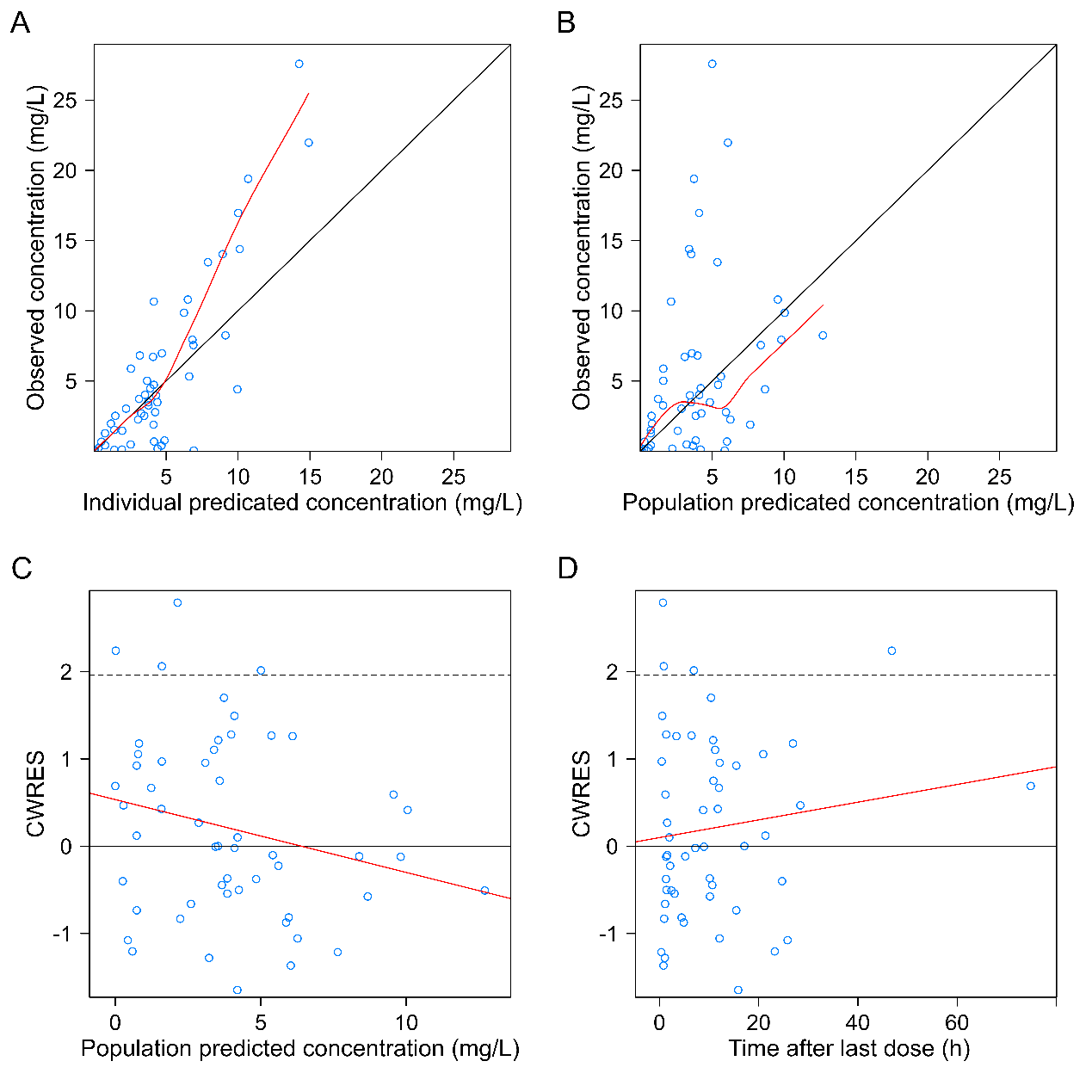


**Figure S2 Diagnostic goodness-of-fit plots of the Nirmatrelvir model.** (A) Observed concentration (DV) vs. individual predicted concentration (IPRED); (B) DV vs. population predicted concentration (PRED); (C) conditional weighted residuals (CWRES) vs. PRED; and (D) CWRES vs. time after last dose. The red lines in the upper panel represent loess smooth lines and linear fit lines, respectively.


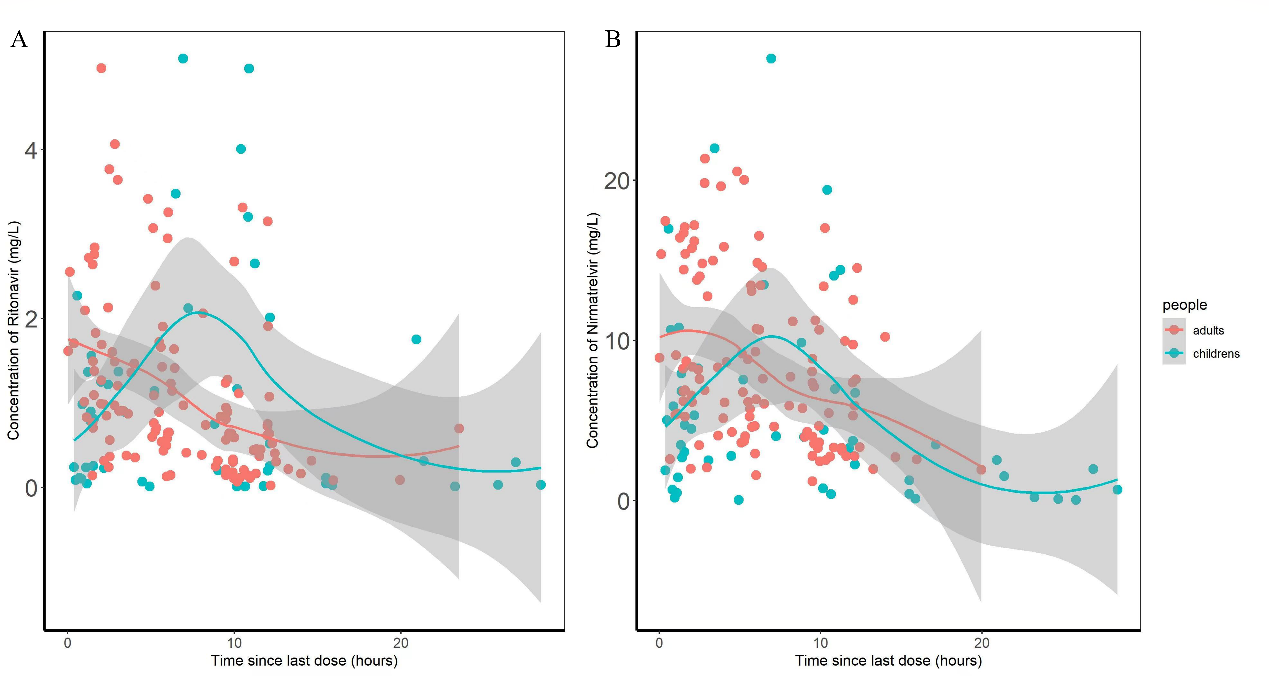


**Figure S3 Serum concentration-time profiles of Nirmatrelvir** (A) and ritonavir (B) in adults and children patients.
